# Supplementary material for: Capsular Polysaccharide Is a Receptor of a Clostridium perfringens Bacteriophage CPS1
Source: Viruses. 2019 Oct 31;11(11):1002. doi: 10.3390/v11111002 (PMC6893597; doi:10.3390/v11111002)
Supplement: Supplementary file 1 [file viruses-11-01002-s001.pdf]

Supplementary materials

Supplementary Figure

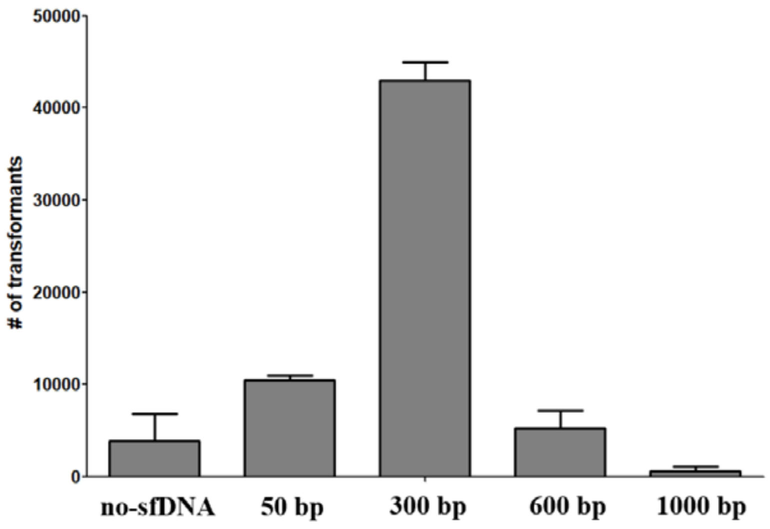

Figure S1. Optimization of *C. perfringens* ATCC 13124 electroporation conditions; The short fragments of DNA (sfDNA) with different sizes including 50 bps, 300 bps, 600 bps, and 1000 bps were tested at 30°C. *C. perfringens* ATCC 13124 was transformed with 1 μg of pJIR750 mixed with 15 pmol of sfDNAs.

Supplementary Tables

Table S1. Primers used in this study

Primer	Sequence (5′-3′)
**CONSTRUCTION OF PLASMID**	
*CPF_0486*_F_BamHI	GCGGGATCCTATTAAACCCATATAAAATAAGAAAGGATATAAA
*CPF_0486*_R_SalI	CGCGTCGACCCTCTTATCCCCTTGTAAATCAAAATTTT
pET28a_*CPF_0486*_F_BamHI	AATTGCGGGATCCATGAATATATTATTAACTGGTGGAATT
pET28a_*CPF_0486*_R_EcoRI	AATTCGCGAATTCTTATTTAAAGTTTTTCATTCCAACGCA
**SEQUENCE CONFIRMATION**	
CPS1_endconfirm_F	TACAAGCAACATCACCGTAAAGTGAAACA
CPS1 endconfirm_R	TGCTCAAGTTCATCAATGCTCTTAGCTT
CPS1_resistant_mutant_F	GCCAACGACTACGCACTAGCCAAC
CPS1_resistant_mutant_R	AGATTGTACTGAGAGTGCACCATAT
*CPF_0486*_confirm_F	TGGCTTATAACTTTGGAGGAAGA
*CPF_0486*_confirm_R	ACCATCAGCCACCTTTGAAACAT
pJIR750_seq_F	AGCGGATAACAATTTCACACAGGAAACAGCT
pJIR750_seq_R	AGATTGTACTGAGAGTGCACCATAT

Table S2. Bacterial strains and plasmids used in this study

Strain and plasmid	Genotype and main characteristics
Clostridium perfringens	
FD1	*Clostridium perfringens* FD1; wild-type strain
ATCC 13124	*Clostridium perfringens* ATCC 13124; wild-type strain; host for phage CPS1
ATCC 13124 *CPF_0486*::Tn5	ATCC 13124 with transposon insertion in *CPF_0486*
ATCC 13124 *CPF_0486*::Tn5 + pJIR750::*CPF_0486*	*CPF_0486*::Tn5 complemented with ATCC 13124 *CPF_0486* gene
Escherichia coli	
E. coli TOP10	F- *mcrA* Δ(*mrr-hsdRMS-mcrBC*) φ80*lacZ*Δ*M15* Δ*lacX74 nupG recA1 araD139* Δ(ara-leu)7697 *galE15 galK16 rpsL*(*StrR*) *endA1* λ-
E. coli BL21	*E. coli* BL21, host of inducible recombinant protein expression
**Plasmid**	
pJIR750	*E. coli* - *C. perfringens* shuttle vector, Cm R
pCPF_*0486*	pJIR750::*CPF_0486*; CmR
pET28a_*CPF_0486*	pET28a with ATCC 13124 *CPF_0486* cloned at BamHI/SalI site, encoding His tag at N-terminal domain; Kanr

Table S3. Host range of phage CPS1

**Bacterial strain**	**CPS1 plaque formation**[a]
***Clostridium* strains**	
Clostridium perfringens H3	**I**
Clostridium perfringens H9	**-**
Clostridium perfringens FD-1	**-**
Clostridium perfringens ATCC 3624	**I**
Clostridium perfringens ATCC 13124	+
Clostridium perfringens FORC25	**-**
Clostridium histolyticum ATCC 19401	**-**
Clostridium indolis ATCC 25771	**-**
**Other Gram positive bacteria**	
Listeria monocytogenes EGD-e	**-**
Bacillus cereus ATCC 13061	**-**
Bacillus cereus ATCC 10987	**-**
Bacillus subtilis ATCC 23857	**-**
Staphylococcus aureus RN4220	**-**
Staphylococcus aureus Newman	**-**

[a]+, Presence of plaques; -, absence of plaques; I, inhibition zone.
